# Relapsing Kikuchi-Fujimoto Disease Requiring Prolonged Steroid Therapy

**DOI:** 10.1155/2019/6405687

**Published:** 2019-03-07

**Authors:** Ulrich Gerwig, Rolf Guenter Weidmann, Gregor Lindner

**Affiliations:** Department of General Internal Medicine & Emergency Medicine, Hirslanden, Klinik Im Park, Seestrasse 220, 8027 Zurich, Switzerland

## Abstract

We report the case of a 26-year-old woman with an eight-week history of painfully enlarged cervical lymph nodes, recurrent headache, and malaise. Her medical history was unremarkable. The physical examination showed multiple enlarged cervical lymph nodes. Laboratory examination was unremarkable, and magnetic resonance tomographic imaging showed multiple enlarged cervical lymph nodes with aspect of a lymphoma. Lymph node biopsy revealed Kikuchi-Fujimoto disease, histologically characterized by histiocytic necrotizing lymphadenitis. A therapeutic trial with nonsteroidal anti-inflammatory drugs (NSAID) showed no effect, so steroid therapy was started. Due to relapse of symptoms after steroid withdrawal the tapering regimen was prolonged for a total of seven months.

## 1. Introduction

Kikuchi-Fujimoto disease (KFD) is a normally self-limiting, benign, rare disease. It is also known as histiocytic necrotizing lymphadenitis and is characterized by cervical lymphadenopathy and fever. First reported by Kikuchi and Fujimoto in 1972 as a lymphadenitis in which histology reveals a focal proliferation of histiocytic cells and abundant nuclear debris and absence of neutrophiles and eosinophiles [1]. Despite many studies and case reports in the literature, reported especially by pathologists and otolarygologists, the cause of KFD remains uncertain. It has not been reported in the emergency medicine literature. Most general practitioners and emergency physicians are likely to be unfamiliar with this condition. It has potential for a protracted course with multiple emergency department visits with inappropriate and potentially harmful treatments, when misinterpreted as lymphoma or systemic lupus erythematodes (SLE).

## 2. Case Presentation

A 26-year-old female patient presented herself to our emergency department due to malaise, headache, and right-sided cervical lymphadenopathy for approximately eight weeks. Previous laboratory diagnostics brought by the patient included a negative serology result for Epstein-Barr-Virus (EBV), Cytomegalovirus (CMV), and Human Immunodeficiency Virus (HIV). Symptomatic therapy with mefenamic acid brought only mild release. The patient reported an uncomplicated bite without any signs of an infection by her parrot four months prior to the start of symptoms. Previous medical history revealed no significant medical illnesses and surgical history was positive for breast augmentation surgery only. Family history was negative and the patient reported no travels outside Switzerland recently. The patient had no regular medication and no illicit drug abuse was reported.

The patient presented in good general state of health with subfebrile temperatures and cardiopulmonary vital parameters were in range. Physical examination showed a right-sided cervical lymphadenopathy ranging from the mandibular angle to the clavicle. On the left side an enlarged lymph node was palpated ventral of the M. sternocleidomastoideus. The lymphadenopathy was tender to palpation. The remainder of the physical examination was unremarkable.

C-reactive protein (CRP) level was slightly elevated at 9 mg/L, just as the erythrocyte sedimentation rate (ESR) at 28 mm/h. Other laboratory results, including differential blood count, serum electrolytes, renal retention parameters, liver enzymes, and lactate dehydrogenase (LDH), were all in normal range. Serum protein electrophoresis was compatible with an inflammatory reaction. Further laboratory examinations, including serology results, are given in Table 1.

**Figure 1:**
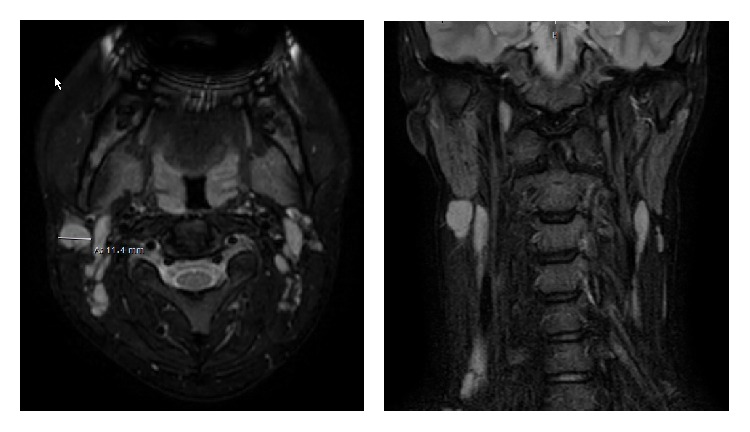
MRI.

Further diagnostics were performed including pharyngeal swabs which were negative. Sonography of the abdomen revealed a slight enlargement of the spleen (12cm). MRI showed a significant enlargement of the cervical lymph nodes (Figure 1).

We suspected a rheumatologic disease causing the lymphadenopathy—thus ANA, ANCA, was performed. ANA titer was elevated at 1:320. Anti-ds-DNA was negative. Considering the clinical presentation of the patient was oligosymptomatic for collagenosis, together with inconclusive laboratory results, we decided to perform a biopsy of the enlarged lymph nodes.

**Figure 2:**
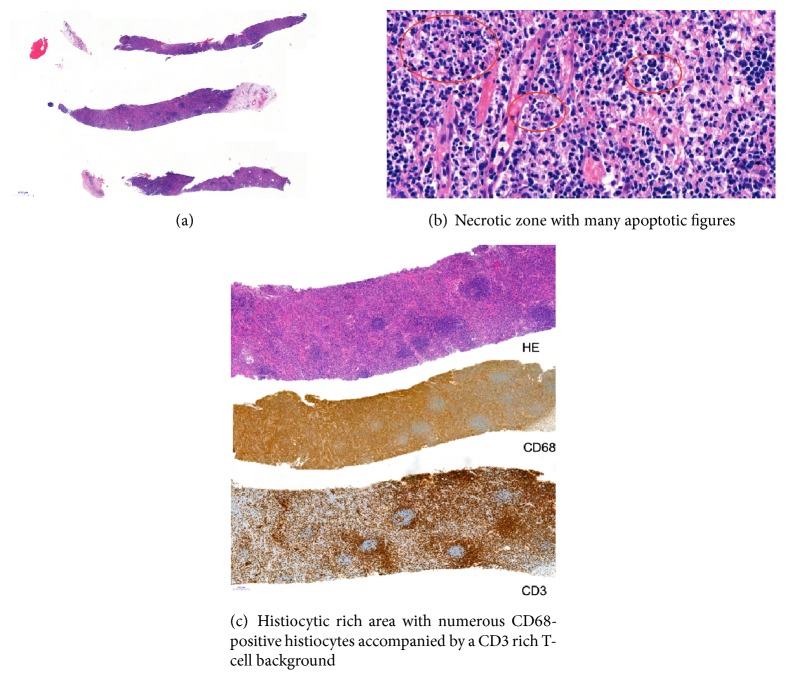


The core needle biopsy revealed signs of a necrotizing lymphadenitis rich in histiocytes. Additional immunophenotypical and molecular analysis underpinned the reactive nature of this lesion (Figures 2(a)–2(c)). The differential diagnosis was raised between KFD and an autoimmune disease of SLE type.

As the patient did not meet diagnostic criteria by the American College of Rheumatology for SLE we suspected KFD.

Consequently, we started a therapy with NSAIDS, resulting in no improvement of the patients' malaise and no regression of the lymphadenopathy. Subsequently, we established steroid therapy at a dose of 1 mg/kg body weight for seven days, followed by a tapering regimen.

After establishment of the steroid therapy, a quick remission of the lymphadenopathy was achieved and the malaise of the patient was resolved.

Due to a three-time relapse of symptoms after steroid withdrawal, the tapering regimen was prolonged for a total of seven months.

After having been without symptoms for approximately 1 year, a relapse of symptoms for some weeks with another episode of contralateral cervical lymphadenopathy occurred, which was again successfully treated with a short steroid taper. The patient has been without symptoms since then.

## 3. Discussion

Kikuchi-Fujimoto disease is a usually benign, mostly self-limited, rare cause of lymphadenopathy. The etiology has remained unknown since its first description by the Japanese pathologists Kikuchi and Fujimoto in 1972 [1]. Associations between SLE and other autoimmune diseases and KFD have been reported [2]; about 20% of cases of SLE are associated with KFD [3]. There is a female predominance as well as a preferential involvement during the third decade of life. However it had been reported in all races that the patients are commonly of Asian descent.

The clinical picture of KFD is similar to that of viral infection. It typically presents as painful cervical, mostly unilateral lymphadenopathy. Less common manifestations are axillary and mesenteric lymphadenopathy, arthralgia, myalgia, splenomegaly, low grade fever, aseptic meningitis, and interstitial lung disease. Except for elevated CRP, ESR, and lymphopenia in about half of patients, laboratory examination does not aid in the diagnosis [3].

Diagnosis is based on the histopathological findings of a lymph node biopsy, which typically shows a histiocytic necrotizing lymphadenitis. Fine-needle aspiration is unreliable [4].

Differential diagnoses of KFD include infectious lymphadenitis (Brucella, Yersinia, HIV, tuberculosis, herpes virus, Epstein-Barr virus, hepatitis B, cytomegalovirus, and parvovirus B19), parasites (toxoplasmosis), autoimmune lymphadenopathy (primarily SLE lymphadenopathy), lymphoma, and lymph node metastasis [5].

KFD is typically self-limiting with resolving of the symptoms spontaneously within a few months. It has a low recurrence rate of 3% to 4% [6]. Supportive measures as NSAIDS and analgesics may be used to alleviate lymph node tenderness and fever. When treatment is necessary, short duration oral corticosteroid therapy is the treatment of choice [5]. Patients with relapsing disease as in our case, or a more severe clinical course, might benefit from a prolonged corticosteroid therapy [6].

## Figures and Tables

**Table 1:** Laboratory values.

Hemoglobin Platelet count Leukocytes INR aPTT Sodium Potassium Calcium ASAT ALAT GGT AP Bilirubin LDH Creatinine Urea CRP ESR Creatine kinase ACE Β-2 microglobulin TSH	120-154 g/l 150-370 G/l 3.9-10.2 G/l < 32s 132-146 mmol/l 3.5-5.0 mmol/l 2.1-2.6 mmol/l < 35 U/l < 35 U/l < 40 U/l 35-104 U/l 3.1-18.6 *µ*mol/l 117-213 U/l 45-84 *µ*mol/l 2.6-6.7 mmol/l < 5.0 mg/l < 20 mm/h < 170 U/l 20-70 U/l < 3.0 mg/l 0.2-4.0 mU/l	117 394 4.55 1.0 42 140 4.2 2.37 20 22 12 67 5 150 53 4.0 9.4 28 47 17 2.6 2.081	Chlamydia psittaci IgG Chlamydia psittaci IgM CMV IgG CMV IgM CMV quantitative, DNA EBV VCA IgM EBV VCA IgG EBV EBNA IgG Epstein-Barr-Virus, DNA HSV-1 IgG HSV-2 IgG HSV-1+2 IgM Syphilis screening Parvovirus B19 IgG Parvovirus B19 IgM VZV IgG VZV IgM Borrelia burgdorferi IgG Borrelia burgdorferi IgM Anti-ds-DNA-antibody ANA ANCA	<1:16 Titer <1:10 Titer <1.0 MOC <1.0 MOC < 1.0 MOC <1.0 MOC <1.0 MOC <1.0 MOC <1.0 MOC <10 AU/ml <0.9 Index <10 U/ml <1:160 Titer <1:20 Titer	<1:16 <1:10 negative negative negative negative positive positive negative 3.6 0.3 0.3 negative 7.8 <0.8 2.9 <0.8 <5.0 <0.64 <0.5 1:320 1:20
